# Meiotic Recombination Defects and Premature Ovarian Insufficiency

**DOI:** 10.3389/fcell.2021.652407

**Published:** 2021-03-08

**Authors:** Chengzi Huang, Ting Guo, Yingying Qin

**Affiliations:** ^1^Center for Reproductive Medicine, Cheeloo College of Medicine, Shandong University, Jinan, China; ^2^National Research Center for Assisted Reproductive Technology and Reproductive Genetics, Shandong University, Jinan, China; ^3^Key Laboratory of Reproductive Endocrinology of Ministry of Education, Shandong University, Jinan, China; ^4^Shandong Provincial Clinical Medicine Research Center for Reproductive Health, Shandong University, Jinan, China

**Keywords:** premature ovarian insufficiency, meiosis, homologous recombination, mutations, next-generation sequencing

## Abstract

Premature ovarian insufficiency (POI) is the depletion of ovarian function before 40 years of age due to insufficient oocyte formation or accelerated follicle atresia. Approximately 1–5% of women below 40 years old are affected by POI. The etiology of POI is heterogeneous, including genetic disorders, autoimmune diseases, infection, iatrogenic factors, and environmental toxins. Genetic factors account for 20–25% of patients. However, more than half of the patients were idiopathic. With the widespread application of next-generation sequencing (NGS), the genetic spectrum of POI has been expanded, especially the latest identification in meiosis and DNA repair-related genes. During meiotic prophase I, the key processes include DNA double-strand break (DSB) formation and subsequent homologous recombination (HR), which are essential for chromosome segregation at the first meiotic division and genome diversity of oocytes. Many animal models with defective meiotic recombination present with meiotic arrest, DSB accumulation, and oocyte apoptosis, which are similar to human POI phenotype. In the article, based on different stages of meiotic recombination, including DSB formation, DSB end processing, single-strand invasion, intermediate processing, recombination, and resolution and essential proteins involved in synaptonemal complex (SC), cohesion complex, and fanconi anemia (FA) pathway, we reviewed the individual gene mutations identified in POI patients and the potential candidate genes for POI pathogenesis, which will shed new light on the genetic architecture of POI and facilitate risk prediction, ovarian protection, and early intervention for POI women.

## Introduction

Premature ovarian insufficiency (POI) is the depletion or dysfunction of ovarian follicles before the age of 40, which is characterized by menstrual disturbance (amenorrhea or oligomenorrhea) for at least 4 months, with raised gonadotrophins (FSH > 25 IU/I on two occasions > 4 weeks apart) and estrogen deficiency (European Society for Human et al., 2016). Approximately 1–5% of women under 40 years old are affected by POI, demonstrated with isolated or syndromic phenotype (Desai and Rajkovic, 2017). The etiologies of POI are heterogeneous, including genetic factors, autoimmune diseases, infection, iatrogenic factors, and environmental toxins. However, most of the cases are still unexplained, known as idiopathic POI. Genetic defects account for approximately 20–25% of POI patients, including chromosomal abnormalities (10–15%) and monogenic mutations (Qin et al., 2015; Jiao et al., 2017). Until now, more than 75 genes have been found to cause POI, which were involved in various processes, including gonadal development, meiosis, DNA damage repair, follicle development, hormone metabolism, and mitochondrial function (Patino et al., 2017; Franca and Mendonca, 2020). Recently, advances in next-generation sequencing (NGS) allow more identification in DNA damage repair genes. Most of the newly identified genes play predominate roles in meiotic homologous recombination (HR), such as *STAG3* (Xiao et al., 2019), while other genes, although participating in DNA damage repair in somatic cells, are found to be essential for meiotic HR as well, such as *MCM8* (AlAsiri et al., 2015) and *BRCA2* (Weinberg-Shukron et al., 2018). Therefore, the role of meiotic HR genes in POI pathogenesis is indispensable.

**FIGURE 1:**
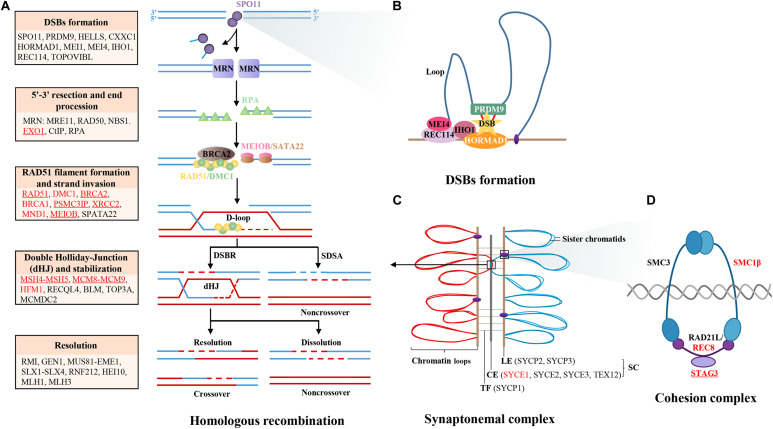
Diagram of meiotic HR genes. **(A)** Major steps of meiotic prophase I, including DSBs formation, 5′-3′ resection, end procession, RAD51 filament formation, strand invasion, intermediates formation, and resolution. The key genes in different steps are labeled in the box, those genes that have been identified in POI patients are labeled with red color, and those that have been functionally validated are underlined. **(B)** The formation of DSBs is initiated by PRDM9, which binds to chromatin and catalyzes H3K4 trimethylation to mark hotspots. Then, the complex MEI4/REC114/IHO1 binds to HORMAD1 on the axis and activates SPO11 to cut chromatin to form DSBs. **(C)** The synapsis complex is installed by CE and LE with TF connections in each pair of homologous chromosomes, which establish the platform of HR. **(D)** The cohesion complex regulates sister chromatid cohesion and SC formation, which is consisted of meiosis-specific subunits STAG3, RAD21L, and SMC1B and non-specific subunits SMC3 and REC8. Notes: DSBs, double-strand breaks; SC, synaptonemal complex; LE, lateral element; CE, central element; TFs, transverse filaments; HR, homologous recombination.

Females are born with fixed number of oocytes within the ovaries. The fertile lifespan depends on the size of oocyte pool at birth and the rapidity of follicle depletion. The initial oocyte pool is determined by the number of primordial germs cells migrating to the genital ridge, followed by germ cell proliferation and functional meiosis, established as the number of primordial follicles at puberty. The human germ cells enter into meiosis from week 9 postconception, go through leptotene, zygotene, and pachytene, and then transitorily arrest at diplotene stage from the time of birth until puberty when primordial follicles are activated and meiosis continues secondary to FSH and LH secretion. During meiotic prophase I, the key processes are deliberate generation of DNA double-strand breaks (DSBs) and subsequent HR, which laid the foundations of stability and diversity of oocyte genome (Handel and Schimenti, 2010). Disturbance of meiotic HR leads to meiosis blocking before diplotene and DSB accumulation. Animal models defective at DSB formation and HR resulted in early exhaustion of follicle pool and infertility, which were similar to the phenotypes of human POI. While only a few genes have been identified with mutations in POI patients, such as *MSH4* (Carlosama et al., 2017) and *MSH5* (Guo et al., 2017), here, we categorized the genes participating in meiotic HR, candidate genes for human POI, and further reviewed the mutations in detail, Which have been identified in POI patients (Figure 1 and Table 1).

**TABLE 1:** Variants of meiosis HR genes identified in POI patients.

Gene	Aliases	Mechanism/function	Mutations found in POI pedigrees	Mutations found in sporadic POI patients	Phenotype	Inheritance	Ovarian phenotypes of mice model	Fertility of mice model
*STAG3*	–	Subunit of cohesion complex	p.F187fs*7 (Caburet et al., 2014)	–	PA	AR	Follicle are exhausted at 6 weeks of age (Winters et al., 2014)	Infertility
			p.S227* (Colombo et al., 2017)					
			p.Y650Sfs*22 (Le Quesne Stabej et al., 2016)					
			p.L490Tfs*10 (He et al., 2018a)					
			p.N98Qfs*2 and p.Y650* (Franca et al., 2019)					
			p.H293_E295del; p.I297_E298insD (Xiao et al., 2019)					
			p.R1018Dfs*14; p.L220R (Heddar et al., 2019)					
*SYCE1*	*POF12*	Central element of SC	p.Q205* (de Vries et al., 2014)	–	PA, SA	AR	Oocyte loss before reproductive age due to synapsis failure (Bolcun-Filas et al., 2009)	Infertility
			Partial region deletion (∼4 kb) (Zhe et al., 2020)					
*SMC1β*	*SMC1L2*	Subunit of cohesion complex	–	p.I221T; p.Q1177L (Bouilly et al., 2016)	PA, SA	Digenicity	Gradually follicle decrease from 4 to 8 weeks of age (Takabayashi et al., 2009)	Infertility
*REC8*	*–*	Subunit of cohesion complex	–	p.Q154R; p.R300L (Bouilly et al., 2016)	PA, SA	Di-genicity	Completely lack of oocytes at postnatal day 5 (Xu et al., 2005)	Infertility
*RAD51*	*FANCR*	Strand invasion	–	p.E68G (Luo et al., 2020)	PA	AD	–	Embryonic lethal (Tsuzuki et al., 1996)
*DMC1*	*LIM15*	Strand invasion	p.D36N (He et al., 2018b)	p.M200V (Mandon-Pepin et al., 2008)	SA	AR	Early oocyte exhausted in the adult ovaries (Pittman et al., 1998)	Infertility
*FANCU*	*XRCC2*	Strand invasion	p.L14P (Zhang et al., 2019)	–	SA	AR	Gradually follicles loss from postnatal days 21 to 180 (Yang et al., 2018)	Impaired fertility
*PSMC3IP*	*HOP2*	Strand invasion	p.Y163* (Al-Agha et al., 2018)	p.R166A*fs*; p.L144* (Yang et al., 2019a)	PA	AR; AD	Absent follicle in the knockout ovaries (Petukhova et al., 2003)	Infertility
			p.E201del (Zangen et al., 2011)					
*MND1*	*GAJ*	Strand invasion	Partial region deletion (8.6 kb) (Jolly et al., 2019)	–	–	AR	Absent follicles and CL (Pezza et al., 2014)	Infertility
*BRCA2*	*FANCD1*	DNA DSBs repair	p.V2527* and p.S3231*fs*16* (Weinberg-Shukron et al., 2018)	New variations: p.I3312V; IVS-7T > A (Yilmaz et al., 2016)	PA	AR; AD	Absent follicle in adult ovaries (Connor et al., 1997); increased ovarian tumor incidence (Szabova et al., 2012)	Infertility
			c.68-1G > C and p.Y1480*; p.D2723V and p.C3233Wfs*15 (Qin et al., 2019)					
*BRCA1*	*FANCS*	DNA DSBs repair	–	New variations: p.T1246N; p.R1835Q (Yilmaz et al., 2016)	–	AD	Decreased primordial follicle number (Titus et al., 2013)	Impaired fertility
*MEIOB*	*SPGF22*	DSB repair/meiotic HR	p.T406 = (Caburet et al., 2019)	–	SA	AR	Completely lack of oocytes at postnatal day 2 (Luo et al., 2013)	Infertility
*MSH4*	–	Stabilization of double Holliday junction	p.l743_K785del (Carlosama et al., 2017)	–	SA	AR	Steady follicle loss soon after birth (Kneitz et al., 2000)	Infertility
*MSH5*	*POF13*	Stabilization of double Holliday junction	p.D487Y (Guo et al., 2017)	p.L353M; p.D487Y; p.I703V (Guo et al., 2017)	SA	AR; AD	Gradually follicle loss after birth and completely devoid at 2–3 months of age (de Vries et al., 1999)	Infertility
				p.P29S (Mandon-Pepin et al., 2008)				
*MCM8*	*POF10*	HR intermediate process	p.P149R (AlAsiri et al., 2015)	p.H317L; p.H601R (Dou et al., 2016)	PA, SA	AR; AD	Follicles were absent at 8 weeks post-partum; ovarian adenomas and sex cord stromal tumors developed in older females (Lutzmann et al., 2012)	Infertility
			p.H161P (Bouali et al., 2017)	p.C155Y; p.N183S; p.R445Q (Desai et al., 2017)				
			c.1954-1G > A; p.L491Ifs*88 (Tenenbaum-Rakover et al., 2015)					
			p.K118Efs*5 (Zhang et al., 2020)					
			p.R309* (Heddar et al., 2020)					
*MCM9*	*ODG4*	HR intermediate process	P.R132*; c.1732+2T > C (Wood-Trageser et al., 2014)	p.T139A; p.T595R; p.V808I; p.Q551*; p.E670* (Desai et al., 2017)	PA, SA	AR; AD	Completely devoid of oocytes in adult ovaries (Lutzmann et al., 2012)	Infertility
			p.E495* (Fauchereau et al., 2016)	p.L475F; p.L974S; p.A1130T (Guo et al., 2020)				
			p.E225Kfs*4 (Goldberg et al., 2015)	p.T595R; c.905-1G > T (Yang et al., 2019a)				
			p.Q551* (Desai et al., 2017)					
*HFM1*	*POF9*	Crossover formation and proper synapsis	P.C1157Y (Zhe et al., 2019)	p.H414P; p.R1194C (Pu et al., 2016)	SA	AD; AR	Follicles are almost exhausted at 45 days (Guiraldelli et al., 2013)	Infertility
			p.I884S and c.1686-1G > C (Wang et al., 2014)	p.G736S and p.P1310Rfs*41 (Wang et al., 2014)				
*EXO1*	*HEX1*	Crossover resolution	–	p.T52S (Luo et al., 2020)	PA	AD	Small ovary with oocytes loss at 7 months of age (Wei et al., 2003)	Infertility
*FANCM*	*FAAP250*	HR intermediate process	p.Q1701* (Fouquet et al., 2017)	–	SA	AR	Depleted primary follicles and reduced developing follicles in ovaries (Bakker et al., 2009)	–
*FANCL*	*FAAP43,PHF9*	–	–	p.Q350Vfs*18; p.M247Nfs*4 (Yang et al., 2020)	PA, SA	AD	Reduced follicles at 4 weeks, germ cell-deficient (GCD) phenotype (Agoulnik et al., 2002)	Infertility
*FANCA*	*FAA*	–	–	p.R591Q; p.E1296G (Yang et al., 2019b)	PA, SA	AD	Significantly reduced follicles and obvious hypogonadism (Cheng et al., 2000; Wong et al., 2003)	Impaired fertility
*SPIDR*	–	HR intermediate process	p.W280* (Smirin-Yosef et al., 2017)	–	PA	AR	–	–
*NUP107*	–	Meiosis/DNA repair	p.R355C (Ren et al., 2018); p.D447N (Weinberg-Shukron et al., 2015)	–	PA	AR	–	–

DSBs, double-strand breaks; HR, homologous recombination; SC, synaptonemal complex; AD, autosomal dominant; AR, autosomal recessive; PA, primary amenorrhea; POI, premature ovarian insufficiency; SA, secondary amenorrhea; CL, corpora lutea; “–” unknown. “*”means “stop codon”.

## Subsections Relevant for the Subject

### Programmed Double-Strand Break Formation

At the beginning of meiotic prophase I, accurate DSB localization and formation are the basis of homologous chromosome recognition and synapsis, and indispensable for crossover, which is crucial for chromosome segregation and formation of euploid gametes. The predominant protein determining potential DSB sites is PRDM9, which recognizes the DSB hotspots on the chromosome loops, catalyzes H3K4 trimethylation (Sun et al., 2015; Chen et al., 2020), and binds to the chromosome axis through interaction with protein CXXC1, HORMAD1 (Daniel et al., 2011), MEI4 (Kumar et al., 2010), REC114 (Kumar et al., 2018), and IHO1 (Stanzione et al., 2016; Kumar et al., 2018). Then, HELLS and PRDM9 form a pioneer complex to open chromatin at hotspots, permitting correct placement and repair of DSBs (Spruce et al., 2020). Then, the endonuclease SPO11 is recruited at PRDM9-binding sites before or after the loop axis interaction and catalyzes DSB formation at the hotspots (Romanienko and Camerini-Otero, 2000). Moreover, other proteins required along with SPO11 to generate DSBs including MEI1 (Reinholdt and Schimenti, 2005) and TOPOVIBL (Robert et al., 2016). The knockout mouse models of the above genes demonstrate female infertility and premature depletion of oocytes due to defective DSB formation and homologous synapsis, except for CXXC1 and HORMAD1. The conditional knockout mice of *Cxxc1* are fertile (Tian et al., 2018). *Hormad1* deficiency does not affect folliculogenesis but disrupts homologous chromosome segregation, resulting in infertility due to gemmate aneuploidy (Shin et al., 2010). Although most of the genes involving DSB formation might be candidate genes for human POI, no causative mutation has been identified in POI patients. Interestingly, there are findings that bi-allelic deleterious mutations of *TOROVIBL*, *MEI1*, and *REC114* could result in recurrent androgenetic complete hydatidiform moles due to extrusion of all maternal chromosomes with the spindles into the first polar body during meiosis metaphase I. These findings indicated that DSB-formed genes were essential for stabilization of gemmate genome. Defects in these genes might be pleiotropic and responsible for heterogeneous reproductive phenotypes (Nguyen et al., 2018).

### DSB End Processing

After DSB formation, DNA ends are engaged in a process of maturation, involving the release of SPO11-oligonucleotide covalent complexes and exonucleolytic degradation on the same strand, which leads to extended overhanging of 3’ single strands on both sides of the DSBs. This process is facilitated by the MRN complex, EXO1, CtIP, and RPA.

The multiprotein complex MRN is consisted of MRE11, RAD50, and NBS1, which are evolutionarily conserved in 5′-end resection of DSBs (Anand et al., 2019). Disruption of the N-terminal exons of *Nbs1* in mice resulted in female infertility due to oogenesis failure (Kang et al., 2002). The female mice with a mutation in *Mre11* exhibited premature oocyte elimination attributing to defects in homologous chromosome pairing and DSB repair during meiotic prophase I (Inagaki et al., 2016). *Rad50* heterozygous mutant mice demonstrated ovarian atrophy as well (Roset et al., 2014). These animal models indicated the essential role of the MRN complex in maintenance of the primordial follicle pool. In humans, mutations in MRN subunits caused Nijmegen Breakage Syndrome in recessive pattern, in which POI was one of the degenerative changes (Chrzanowska et al., 2012). Although they are potential causative genes for POI, no mutation has been identified in isolated POI yet. Besides that, CtIP is an important cofactor of MRN in catalyzing the 5′-end resection (Sartori et al., 2007). *CtIP* mutations cause Seckel and Jawad syndromes in a recessive manner, while no ovarian abnormality was noticed (Qvist et al., 2011). Therefore, although CtIP performs an important role in DSB end processing, it might not be a potential causative gene of POI.

EXO1 has 5′ to 3′ exonuclease activity, which is recruited to DSBs by MRN and promotes the formation of 3′-tailed single-strand DNA (ssDNA) (Garcia et al., 2011). *Exo1* knockout female mice were infertile due to dynamic loss of chiasmata during meiosis prophase I (Wei et al., 2003). A meta-analysis of 53 GWASs with nearly 70,000 women found EXO1 polymorphism associated with the age of natural menopause (Day et al., 2015). Recently, through whole-exome sequencing (WES) in 50 patients with POI, one heterozygous mutation in *EXO1* was identified, which impaired meiosis by disrupting recruitment of RPA and RAD51 onto DSB sites (Luo et al., 2020). These findings confirmed the role of EXO1 in POI, and discussed the dosage-dependent effect of oocyte-non-specific HR gene on ovarian function.

When 3′-tailed ssDNAs are established, RPA is recruited to prevent ssDNA degradation or formation of secondary structure (Soustelle et al., 2002). Recent study found that the loss of RPA completely abrogated the loading of recombinases RAD51 and DMC1 on DSBs sites, blocked strand invasion, and chromosome synapsis (Shi et al., 2019). However, because RPA is ubiquitously expressed, the *Rpa1* null mice showed embryonic lethality. Although the heterozygotes displayed defects in DSB repair, ovarian phenotype had not been observed (Wang et al., 2005). Therefore, the evidence of RPA participating in POI pathogenesis was insufficient yet.

### Strand Invasion

As the proceeding of HR, RPA is replaced by the recombination proteins RAD51 and DMC1, which catalyze homology search and strand invasion, establishing the basis of synapsis (Brown and Bishop, 2014). Besides RAD51 and DMC1, the dynamic process is also regulated by other recombination factors, including BRCA2, PSMC3IP, MND1, MEIOB, and SPATA22.

RAD51 and its meiotic paralog DMC1 execute the critical step of strand invasion (Bishop et al., 1992; Park et al., 2008). In the *Dmc1*-deficient mice, gametogenesis arrested in meiotic prophase I, resulting in germ cell depletion in the adult ovaries and infertility. Recently, a homozygous mutation p.D36N in *DMC1* was identified in one consanguineous pedigree having one patient with POI and one patient with non-obstructive azoospermia (NOA) (He et al., 2018b). Histological study found that spermatogenesis was blocked at zygotene stage in the patient with NOA, indicating that POI in women might be caused by dysfunctional meiosis prophase I of oocytes (Pittman et al., 1998). Besides that, another homozygous mutation *DMC1* p.M200V was identified by Sanger sequencing in sporadic POI (Hikiba et al., 2008), while the point mutant mice showed normal ovarian morphology, highlighting the importance of functional studies in verifying the pathogenicity of variations (Hikiba et al., 2008; Tran and Schimenti, 2018). Absolutely, loss of RAD51 resulted in embryo lethality in mice (Tsuzuki et al., 1996). However, a WES study in sporadic POI patients found one heterozygous missense mutation of *RAD51*, which resisted the protein localization in the nucleus. *In vitro* experiments found that heterozygous mutation affected HR efficiency by haploinsufficiency, indicating that the pathogenic effect of *RAD51* on POI might be dosage dependent (Luo et al., 2020).

BRCA2 regulates the localization of RAD51 onto ssDNA to form an RAD51-ssDNA filament, promoting HR repair for DSBs both in somatic cells and in germ cells (Davies et al., 2001; Xia et al., 2001). Somatic *BRCA2* mutations impaired chromosome integrity, manifesting with an increased risk of tumor (Daum et al., 2018), whereas recent studies found its crucial role in ovarian development mediated by functional meiotic recombination (Miao et al., 2019). Until now, four pairs of compound heterozygous mutations and one homozygous mutation in *BRCA2* have been identified in POI pedigrees through WES analysis (Weinberg-Shukron et al., 2018; Qin et al., 2019; Caburet et al., 2020). Among them, four mutation carriers demonstrated with microcephaly, leukemia, thyroid cancer, or breast carcinoma, while other three carries presented with isolated POI. The widely varying severity of clinical profiles of bi-allelic *BRCA2* mutation carriers confirmed the complicated function of BRCA2, also highlighted the necessity of long-term follow-up for them. Recent studies in *Caenorhabditis elegans* found BRCA1 influenced RAD51 dynamics and combined with SYCP3 and MSH5 to promote synapsis and crossover resolution (Janisiw et al., 2018). Although its function in mammalian meiosis was unclear, *Brca1* mutant mice had impaired reproductive capacity and decreased primordial follicle counts (Titus et al., 2013). Women with *BRCA1* variations also presented with accelerated ovarian reserve decline (Lin et al., 2017; Porcu et al., 2020). Therefore, BRCA1 was a potential causative gene of POI, which required comprehensive evaluation of somatic characteristics like BRCA2.

PSMC3IP (also known as HOP2) and MND1 are meiosis-specific factors in all organisms expressing DMC1. PSMC3IP-MND1 complex facilitates strand invasion and D-loop formation by promoting DMC1/RAD51 capturing of double-strand DNA (dsDNA) (Chi et al., 2007; Pezza et al., 2007). Absence of them resulted in non-homologous synapses and DSB accumulation (Sansam and Pezza, 2015). *Psmc3ip* and *Mnd1* knockout mice showed severely reduced ovarian size and defective gametogenesis (Petukhova et al., 2003; Pezza et al., 2014). In previous studies, two-point mutations of *PSMC3IP* and one microdeletion of *MND1* inherited in recessive patterns have been identified in consanguineous pedigrees with POI or XX female gonadal dysgenesis (Zangen et al., 2011; Zhao and Sung, 2015; Al-Agha et al., 2018; Jolly et al., 2019), confirming their crucial roles in gametogenesis and POI pathogenesis.

MEIOB and SPATA22 are ssDNA-binding proteins predominately expressed in meiosis prophase I, which form a complex that interacts with RPA to recruit RAD51 and DMC1 to the ssDNA (La Salle et al., 2012; Luo et al., 2013; Souquet et al., 2013; Ishishita et al., 2014). Both *Meiob*-null mice and *Spata22*-null mice exhibited small ovaries devoid of oocytes in any developmental stage due to uncompleted meiotic HR (Luo et al., 2013; Hays et al., 2017). Recent WES study with a POI pedigree identified one homozygous splicing mutation in *MEIOB*, which resulted in a truncated MEIOB protein, thus interrupting the interaction with SPATA22 (Caburet et al., 2019). POI might be caused by defective MEIOB-SPATA22 complex-induced insufficient DNA single-strand invasion during meiotic HR. Although no mutation has been found in *SPATA22*, it still is a potential candidate gene of POI.

### Intermediate Processing and Homologous Recombination

During strand invasion, the presynaptic filaments recognize the template strands, invade into the duplex DNA, displace the original strand, and bind to their complementary sequence, forming the intermediate of HR repair. The intermediate processing is performed by two pathways: synthesis-dependent strand annealing and double Holliday junction (dHJ). SDSA is a pathway for non-crossover repair, in which a D-loop intermediate is formed and the broken DNA is synthesized using the homologous chromosome as a template (Ranjha et al., 2018). In the dHJ pathway, two DSB ends participate in the invasion that forms a classic double junction intermediate, which facilitates crossover formation and resolution. During the process, MSH4–MSH5 heterodimer, MCM8–MCM9 helicase complex, HFM1, RECQL4, BLM, and MCMDC2 are involved.

The meiotic specially expressed proteins MSH4 and MSH5 form a heterodimeric complex (Acharya et al., 1996; Snowden et al., 2004), which clamps on homologous chromosomes to stabilize the Holliday junctions (Nishant et al., 2010). In the *Msh5/Msh4* deficient mice, chromosome pairing was failed and crossover was absent, resulting in atrophic ovaries, which were similar to the phenotype of human POI (de Vries et al., 1999; Kneitz et al., 2000). Through WES in two POI pedigrees, homozygous mutations of *MSH4* and *MSH5* were identified, pathogenicity of which was confirmed by *in vitro* studies and knock-in mice models (Carlosama et al., 2017; Guo et al., 2017). These results implied the recessive mode of inheritance for *MSH4* and *MSH5* in POI. Interestingly, four heterozygous mutations of *MSH5* have been reported in sporadic cases, indicating that their effects on meiosis and oogenesis might be dominated or dosage dependent as well (Mandon-Pepin et al., 2008; Guo et al., 2017).

MCM8 and MCM9 form a helicase complex regulating DNA repair and genome integrity both in somatic cells and in germ cells (Nishimura et al., 2012). They not only promote MRN-mediated ssDNA maturation, but also participate in intermediate processing of HR. *Mcm8* or *Mcm9* knockout mice suffered meiosis blocking at prophase I (Lutzmann et al., 2012). Bi-allelic mutations of *MCM8* and *MCM9* have been identified in POI patients with or without familial history (Wood-Trageser et al., 2014; AlAsiri et al., 2015; Goldberg et al., 2015; Tenenbaum-Rakover et al., 2015; Fauchereau et al., 2016; Bouali et al., 2017; Desai et al., 2017). The prevalence of bi-allelic mutations of *MCM9* in sporadic cases was variable among different studies, ranging from 1.6 to 6.1% (Yang et al., 2019a; Guo et al., 2020). However, heterozygous variations were also found in 1.0–4.6% of sporadic POI, making the inheritance pattern of *MCM8/MCM9* in recessive or dominate to be ambiguous (Dou et al., 2016; Desai et al., 2017; Guo et al., 2020). Interestingly, some patients were found to carry digenic heterozygous variants in both *MCM8* and *MCM9* or in *MCM8/MCM9* and other DNA repair genes (Desai et al., 2017). Moreover, researchers observed MCM8 had a dosage-dependent effect on the severity of POI phenotypes. These findings indicated that heterozygous variations of HR genes might establish a genetic background susceptive to DNA damage, which would affect meiosis when additional variations in the related genes or environmental toxin existed (Heddar et al., 2020; Wang et al., 2020). Besides the essential role in meiosis, MCM8 and MCM9 were involved in DNA replication, DNA damage response and cell cycle regulation in somatic cells. Some of the mutation carriers presented with short stature, and an *MCM8* carrier was reported to have pilomatricomas (Heddar et al., 2020). Mitomycin-induced DNA breaks and aberrant metaphases in the patient’s lymphoblastoid cells suggested that the patients carrying *MCM8* or *MCM9* mutations were susceptive to tumor or growth retardation due to impaired DNA repair and genome instability in somatic cells. Therefore, long-term follow-up of cancers for those mutation carriers is needed.

HFM1 is a DNA helicase preferentially expressed in germline cells. Absence of HFM1 resulted in aberrant intermediate processing and reduced crossover formation (Guiraldelli et al., 2013). Two-compound heterozygous mutations of *HFM1* were identified in two familial POI and one sporadic case (Wang et al., 2014). Moreover, heterozygous pathogenic mutations were found in a POI pedigree and 1.5% of the sporadic case, indicating that *HFM1* mutants might cause POI through both recessive and dominate modes (Pu et al., 2016; Zhe et al., 2019).

RECQL4 and BLM are pleiotropic helicases expressed non-specifically, which unwind dsDNA into ssDNA during HR repair for DSBs (Singh et al., 2012). They are essential for maintenance of genome stability in both somatic and germline cells. Therefore, their defects mostly cause syndromic POI, such as Rothmund–Thomson syndrome (Siitonen et al., 2009) and Bloom syndrome (Arora et al., 2014), in which POI is one of the complicated symptoms.

Besides the helicases above, MCMDC2 is an atypical yet conserved MCM protein, which also plays an important role in ssDNA invasion that promotes homolog alignment and inter-homolog crossover formation. *Mcmdc2* knockout mice were infertile, demonstrated to have atrophic ovaries completely devoid of oocyte at 6 weeks post-natal (Finsterbusch et al., 2016). Although no mutation of *MCMDC2* has been reported in POI patient yet, it still is a potential causative gene for POI.

### Synaptonemal Complex and Cohesion Complex

Throughout meiosis prophase I, the chromosomes are reorganized as linear arrays of chromatin loops anchored to a central axis. The chromosome axis forms a platform for the assembly of synaptonemal complex (SC), which plays a central role in homologous pairing, recombination, and chromosome segregation. The SC is installed by five central elements linked to two lateral elements by a transverse filament in each pair of homologous chromosomes.

The central elements of SC include SYCE1-3, C14ORF39, and TEX12. Female knockout mice of those genes were affected by infertility and oocyte loss before reproductive age due to different degrees of synapsis failure (Bolcun-Filas et al., 2007, 2009; Hamer et al., 2008; Schramm et al., 2011; Davies et al., 2012; Lu et al., 2014). In POI patients, except for one microdeletion and two homozygous mutations of *SYCE1* which have been identified (McGuire et al., 2011; Zhen et al., 2013; de Vries et al., 2014; Zhe et al., 2020), no causative mutation has been found in other genes, indicating that the mutations in central elements of SC might not be a common genetic causation for POI.

SYCP1 is the transverse filament of SC that connects central elements SYCE1–2 to the lateral elements localized in each homologous chromosome. Absence of *Sycp1* disturbed chromosomal synapsis, resulting in oocytes arrested at pachytene stage and apoptosis (de Vries et al., 2005). SYCP2 and SYCP3 are lateral elements of SC, which interact with each other (Winkel et al., 2009) and stabilize the linear array of chromatin loops during SC assembly (Yang et al., 2006; Syrjanen et al., 2014, 2017). The *Sycp2* and *Sycp3* mutant mice were subfertile (Yuan et al., 2000; Yang et al., 2006), which might be explained by insufficient SC formation, contrasting to the absolute loss of SC in *Sycp1* null mice (Yang et al., 2006). That dosage-dependent meiosis dysfunction could also be a potential explanation for heterogeneous clinical phenotypes of human POI. Furthermore, *Sycp3* mutant female mice exhibited increased aneuploidy in oocytes and embryos. In human beings, heterozygous variations of *SYCP3* were associated with miscarriage and increased predisposition to infertility (Bolor et al., 2009; Nishiyama et al., 2011). Therefore, although no *SYCP* mutation has been identified in POI patient yet, their roles in oogenesis and embryo development should be further explored.

The cohesion complex regulates sister chromatids cohesion and SC formation, which is composed of meiosis-specific subunits STAG3, RAD21L, and SMC1B and non-specific subunits SMC3 and REC8 (Ishiguro, 2019). Female mice deficient in *Stag3* were sterility and follicle exhausted at a young age (Caburet et al., 2014). To date, seven bi-allelic mutations of *STAG3* have been found in POI pedigrees. All the affected patients manifested with primary amenorrhea and streak ovaries (Caburet et al., 2014; Le Quesne Stabej et al., 2016; Colombo et al., 2017; He et al., 2018a; Franca et al., 2019; Heddar et al., 2019; Xiao et al., 2019), indicating that recessive mutations in *STAG3* were relatively common genetic causation for primary POI. Furthermore, mice deficient in other cohesion genes demonstrated with similar ovarian morphology of *Stag3* null mice (Xu et al., 2005; Takabayashi et al., 2009; Herran et al., 2011). Through target gene screening of sporadic POI patients, heterozygous mutations in *STAG3*, *SMC1B*, and *REC8* have been found (Bouilly et al., 2016), indicating that the recessive and dominate causative modes of cohesion genes in POI might coexist. Moreover, age-dependent decrease of cohesion protein is associated with increased rate of aneuploidy oocytes, while mutations of *SMC3* were reported in Cornelia de Lange syndrome without ovarian abnormalities (Deardorff et al., 2007). Therefore, besides the indispensable contribution to POI, the pleiotropic effects of cohesion genes in reproductive and somatic diseases should be considered as well.

### Resolution of Recombination Intermediates

In germ cells, the essential step for accurate separation of homologous chromosomes at the first meiotic division is resolution of recombination intermediates, including non-crossover pathway and crossover pathway. In the non-crossover pathway, the final products are generated by annealing the invaded strand to the complementary break end of single Holliday junction or dissolution of the dHJs (Bizard and Hickson, 2014; Daley et al., 2014). That process is the major route for dissipation of HR intermediate, which limits chromosomal rearrangements and heterozygosity of oocytes. This reaction requires the RecQ helicase BLM (Wu et al., 2000), topoisomerase TOP3A (Martin et al., 2018), RMI complex (Raynard et al., 2008), structure-selective endonucleases GEN1 (Shah Punatar et al., 2017), MUS81-EME1, and SLX1–SLX4 (Matos and West, 2014; Wyatt and West, 2014). Absence of the above genes resulted in syndromic disease or embryonic lethality. Therefore, their pleiotropic effect on meiosis and ovarian function was illusive and needs more detailed exploration.

Crossover pathway is the meiosis-specific resolution of dHJs that contributes to the genetic diversity of species. Although the process resolves less recombination than non-crossover pathway, the occurrence of at least one crossover in every pair of homologous chromosomes is essential for precise separation of chromosomes in the first meiotic division. Crossover pathway involves *RNF212* (Qiao et al., 2014), *HEI10* (Ward et al., 2007), *MLH1* (Baker et al., 1996), and *MLH3* (Lipkin et al., 2002). The knockout mice of the above genes had normal ovarian morphology; oocytes show proficient synapsis but deficient crossover, presenting with abnormal chromosome alignment at metaphase I and disturbed extrusion of polar bodies. Those female mice were infertile due to a decreased number of mature MII oocytes and increased number of aneuploidy embryos. Therefore, these gene defects are responsible for disorders of oocyte maturation or early embryo development rather than POI.

### Fanconi Anemia Pathway Genes in Meiotic HR

Fanconi anemia (FA) is usually a recessive genetic disease associated with bone marrow failure, increased cancer susceptibility, and severe germline defects. There are 22 identified FA genes, which are involved in DNA interstrand crosslink repair, including the FA core complex which catalyzes the mono-ubiquitination of FANCD2 and FANCI, and DSB repair genes—*BRCA1* (*FANCS*), *BRCA2* (*FANCD1*), *BRIP1* (*FANCJ*), *PALB2* (*FANCN*), *RAD51C* (*FANCO*), *SLX4* (*FANCP*), *RAD51* (*FANCR*), and *XRCC2* (*FANCU*) (Tsui and Crismani, 2019). Although all mice models of FA genes reported to date have different degrees of reduction in fertility, the links between their roles in DNA repair and fertility have not been extensively explained. Recent studies found that BRCA2 promoted the localization of RAD51 and DMC1 to meiotic DSBs. As a member of RAD51 paralogs, FANCU (XRCC2) might be involved in the RAD51-mediated strand invasion during meiotic HR (Yang et al., 2018). Besides that, FA core factors FANCA, FANCB, and FANCC were reported to facilitate the recruitment of FANCD2 on sex chromosomes and regulate the histone modification during meiotic HR (Alavattam et al., 2016). FANCM has also been shown to limit crossover frequencies, which promoted the conservatism of gametes (Crismani et al., 2012). The increasing research of FA genes highlighted their roles in the resolution of meiotic DSBs, giving more indications of oogenesis as well. Up to date, several FA genes had identifications in POI, such as homozygous mutations in *FANCM* (Fouquet et al., 2017) and *FANCU* (Zhang et al., 2019) and heterozygous mutations in *FANCA* (Yang et al., 2019b) and *FANCL* (Yang et al., 2020). Interestingly, heterozygous *FANCA* knockout mice showed a declined follicle number and reduced fertility; *in vitro* studies found that single-allelic defects of *FANCA* and *FANCL* compromised DNA repair ability by haploinsufficiency, indicating that the adverse effects of FA gene variations on meiosis and ovarian function might be dosage dependent.

## Discussion

Identifying causative genes of POI and elucidating their molecular mechanisms are important for the genetic diagnosis of POI. As an increasing number of women prefer to conceive after their mid-30s, the genetic counseling of POI predisposition will be instructive for their childbearing plans. To date, more than 75 genes have been found to be responsible for POI, among which 24 genes were involved in meiotic HR process. With the widespread use of NGS and whole-genome sequencing, the identification of novel genes will be increased in the near future. Furthermore, with the development of data analysis strategies, more non-synonymous mutations with high risk of pathogenicity, microdeletion or interruption, and rearrangement of gene sequences will be identified, expanding the mutation spectrum and genetic architecture of POI.

Along with the increasing genes and variations identified, more challenges are emerging to determine the causative patterns of meiotic HR genes in POI. Most of the meiotic HR genes were found in familial POI by recessive modes, while heterozygous mutations were more common in sporadic cases, and the mutation frequencies varied significantly among different cohorts. These observations indicated that the genetic architecture of sporadic POI would be more complicated than that in familial cases. With more and more di-genetic or multigenetic variations reported and dosage-dependent effect confirmed by functional studies, sporadic POI seemed to be a complex disease, which occurred as a result of multiple genomic variants paired with environmental influences. Furthermore, many HR genes had pleiotropic effects in proliferation and apoptosis of somatic cells. The relationships between pleiotropic genes and heterogeneous phenotypes of isolated or syndromic POI should be further explored as well.

Meiotic HR genes not only participate in oogenesis but also facilitate oocyte maturation, fertilization, and early embryo development. Dysfunction of several genes might be responsible for unexplained infertility or early pregnancy loss, such as members of SC and cohesion complex influenced chromosome separation and aneuploidy of oocytes. Therefore, besides the benefits of early diagnosis, intervention, and treatment of POI, further studies on the meiotic HR genes will give advice to other diseases of infertility and adverse pregnancy outcomes. Moreover, considering the increased cancer susceptibility of HR gene defects, long-term follow-up for cancer risks and healthcare should be suggested.

## Author Contributions

YQ and TG contributed to the conceptual idea. CH performed the data collection. TG and CH wrote the manuscript. YQ reviewed the manuscript. All authors contributed to the article and approved the submitted version.

## Conflict of Interest

The authors declare that the research was conducted in the absence of any commercial or financial relationships that could be construed as a potential conflict of interest.
